# Minireview: Transaortic Transcatheter Aortic Valve Implantation: Is There Still an Indication?

**DOI:** 10.3389/fcvm.2022.798154

**Published:** 2022-03-04

**Authors:** Lukas Stastny, Christoph Krapf, Julia Dumfarth, Simone Gasser, Axel Bauer, Guy Friedrich, Bernhard Metzler, Gudrun Feuchtner, Agnes Mayr, Michael Grimm, Nikolaos Bonaros

**Affiliations:** ^1^Department of Cardiac Surgery, Medical University Innsbruck, Innsbruck, Austria; ^2^Department of Internal Medicine III, Cardiology and Angiology, Medical University of Innsbruck, Innsbruck, Austria; ^3^Department of Radiology, Medical University of Innsbruck, Innsbruck, Austria

**Keywords:** transaortic transcatheter aortic valve implantation, alternative access route for transcatheter aortic valve, TAVI, aortic valve stenosis, technical aspects

## Abstract

Transaortic (TAo) transcatheter aortic valve implantation has become a valid alternative access route in patients with unsuitable femoral arteries. The current literature does not allow to clearly favor one of the alternative access routes. Every approach has its specific advantages. Transaortic (TAo) access is of particular importance in the case of calcifications of the supra-aortic branches and the aortic arch, as under these circumstances other alternative access routes, such as transaxillary or transcarotid, are not feasible. The purpose of this minireview is to give an overview and update on TAo transcatheter aortic valve implantation focusing on indication, technical aspects, and recent clinical data.

## Introduction

Transcatheter aortic valve implantation (TAVI) has become the gold standard in the treatment of severe aortic stenosis in patients who are either unsuitable for surgery or with a high surgical risk (1–3). The reliable results in this challenging patient population have resulted in its expansion to lower-risk patients (4, 5). In randomized controlled trials conducted to prove the safety and efficacy of this therapy, transfemoral (TF) access has been the most widely used access route (6). However, in 5–10% of the patients selected for TAVI, TF access cannot be performed (6, 7). Main causes are the presence of peripheral vascular disease, severe vessel tortuosity, anatomical abnormalities, or calcification in the aortic arch. Consequently, alternative TAVI routes are essential, particularly for inoperable patients (8). Transapical (TA) TAVI was the first alternative access route developed for patients with unsuitable femoral vessels. The procedure is associated with a high risk for bleeding, ventricular damage, myocardial injury, and mortality (9–11). To overcome some of these drawbacks, transaortic access (TAo) was introduced as an additional alternative to TA and TF TAVI. Its usage has been proposed in patients with significant pulmonary disease, severely impaired left ventricular function, or a fragile apex (12, 13). TAo utilizes an upper ministernotomy, which is well tolerated by the patients and a procedure familiar to cardiac surgeons. The puncture and repair of the aorta are routinely performed for cardiopulmonary bypass, and hemostasis can easily be achieved. Furthermore, the crossing of the aortic valve is simplified and positioning of the transcatheter valve prosthesis can be done very precisely (14). Other alternative access (AA) routes, such as transaxillary (TAx) and transcarotid (TC), offer comparable advantages over TA. Several studies have compared these procedures with contrary results in terms of procedural success, perioperative outcome, and mortality (15–19).

The purpose of this minireview is to give an overview and update on TAo TAVI, focusing on indication, technical aspects, and recent clinical data.

## Preprocedural Planning: Indications for Tao Access

In the preprocedural planning of TAVI, several assessments need to be performed to give the multidisciplinary heart team enough information for a sophisticated decision. These investigations include at least: coronary angiography, transthoracic echocardiography, carotid artery duplex scan, lung function test, and multislice computed tomography scan (MSCT). MSCT is the key step for a precise characterization of the anatomy from the aortic root to the ileofemoral vessels (20). Due to the cyclic motion of the heart, ECG-synchronization is crucial to achieving the desired resolution of the aortic valve, the aortic annulus, and the ascending aorta. Furthermore, MSCT provides all essential information for the selection of the access route (21).

While a minimal vessel lumen diameter (>5.5 mm) from the left or right femoral artery to the aortic valve is required to perform a TF TAVI, peripheral artery disease is the most common contraindication in this approach. In addition to a detailed analysis of the iliacofemoral arteries, exploration of the aorta should be carried out to identify possible challenges or contraindications such as tortuosity, presence of an aneurysm, thrombotic appositions, or aortic arch calcifications. In the presence of one of these circumstances, the heart team has to consider alternative access routes; the TAo access can be used in almost all patients. The only relative contraindications are previous cardiac surgery, thoracic deformities, short ascending aorta, and porcelain aorta. Individual calcified sites are not a contraindication, but require precise preoperative planning. In contrast to other alternative access routes, TAo entails no additional learning curve for a cardiac surgeon. Purse-string suture and cannulation of the ascending aorta are standard procedures and are performed daily. Therefore, access site complications are not common in the literature and are described are described in 2 to 5% only (17, 22–24). The TAo access is of particular importance in case of contraindications for TF TAVI and concomitant calcifications of the supra-aortic branches and the aortic arch since under these circumstances other alternative access routes, such as TAx or TC, are not feasible (25).

## Procedure

The procedure is performed under general anesthesia in a hybrid operating room. Fluoroscopy and transesophageal echocardiography are the main imaging technologies during the implantation. The patient is placed in a supine position and is hemodynamically monitored. In general, the operating room setup differs from other TAVI procedures. The implanting surgeon is standing at the head end of the patient. In a first step, a transvenous pacing wire is placed in the right ventricle for rapid pacing, either through the femoral or the jugular vein. An arterial sheath (6-French) is inserted into the femoral or radial artery to place a pigtail catheter into the aortic root (26).

Depending on the position of the ascending aorta, two different types of thoracic accesses are used: upper ministernotomy and mini right thoracotomy in the 2nd intercostal space. The first one is preferred if the ascending aorta is in the midline or toward the left and over 6 cm deep to the sternum. In contrast, mini right thoracotomy is used if the ascending aorta is over 50% on the right side and not more than 6 cm away from the sternum (27).

**Figure 1:**
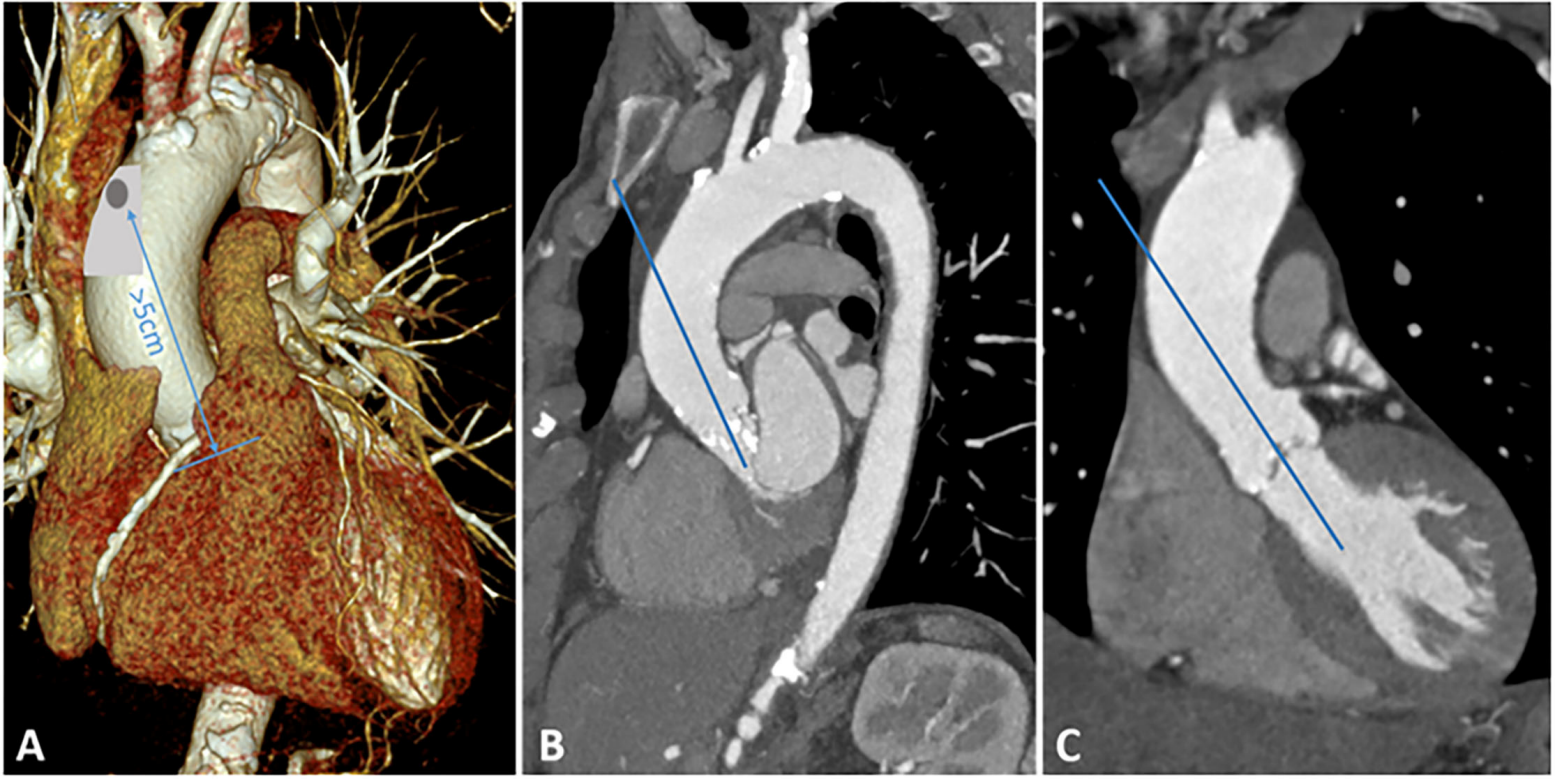
Preoperative MSCT. **(A)** 3D reconstruction of the aortic arch, appropriate location for aortic puncture is highlighted in gray. The distance between the puncture and the aortic annulus should be at least 5 cm. **(B)** Sagittal plane of the aortic arch. **(C)** Coronary plane of the ascending aorta.

After a longitudinal incision of the pericardium, the ascending aorta can be exposed very easily. Identification of a suitable location is crucial: the puncture should be more than 5 cm away from the aortic annulus. In addition, the angle of puncture should be in line with the left ventricular outflow tract. In most cases, the anterolateral part of the ascending aorta is well applicable. Figure 1 illustrates the most appropriate location for aortic puncture to achieve the desired angle in the aortic root. In the next step, the ascending aorta is gently manipulated to identify atherosclerotic deposits, which should be avoided as an entry point. Two circular purse strings are placed on the selected spot with 3-0 prolypropylene.

Heparin is administered with a dose of 100 IU/kg body weight to achieve an ACT of 300 s or above. The ascending aorta is then punctured within the pure-string sutures and a soft guidewire is introduced in the aorta. The needle is replaced by an eight French multipurpose sheath. The crossing of the aortic valve is achieved with a hydrophilic crossing guidewire, which is replaced by a preshaped extra-stiff wire. If necessary, a predilatation is performed. Two different valve types are used for TAo TAVI: self-expandable (Medtronic CoreValve Evolut R/Pro, Medtronic Inc., Minneapolis, MN, USA) and balloon-expandable (Edwards Sapien XT/3, Edwards Lifesciences Corp., Irvine, CA, USA).

## Self-Expandable Valve

After checking whether the wire is at the right angle in the right place, the EnVeo PRO delivery system (18 French, Medtronic Inc., Minneapolis, MN, USA) is introduced. The CoreValve Evolut R is placed in the desired position under fluoroscopy and TEE guidance. Rapid pacing is initiated with 120 beats per minute. The deployment of the valve is done in a stepwise manner. This allows a maximum degree of control and the possibility of repositioning the valve (27).

## Balloon-Expandable Valve

Edwards Lifescience Corp. provides two different delivery systems for the TAo approach: the certitude and the TF delivery system. The Certitude delivery system, which is normally used for the TA approach, is easier to handle because of the shorter length. Additionally, no loading maneuver is necessary. The TF delivery system has some disadvantages: (i) the valve has to be loaded onto the balloon in the ascending aorta, (ii) the length of the delivery system requires a nonstandardized room set-up. Positioning of the valve is controlled by fluoroscopy and TEE. The deployment of the valve is performed under rapid pacing with 200 beats per minute (26).

Regardless of the used valve type, an aortogram must be obtained to confirm the correct transcatheter heart valve (THV) position, to check for paravalvular leakage, and to assess the patency of coronary arteries.

## Literature Overview: Clinical Data

The most common approach for TAVI with the lowest incidence of procedural complication remains the access through the femoral artery (28). However, in 5–10% of patients selected for TAVI, TF access is not feasible. Peripheral artery disease with impassable stenosis, severe tortuosity, or small caliber of the iliofemoral vessel is the main driving factor for alternative access routes (7). The first successful TAo TAVI was conducted in 2010 by Bapat et al. (29) Since then, TAo TAVI has become an accepted alternative approach with satisfactory clinical outcome and acceptable risk.

The ROUTE registry (Registry for Utilization of TAo-TAVI Approach Using the Edwards SAPIEN Valve), a multicenter, prospectively collected, European registry has reported on 301 high-risk patients who underwent TAo-TAVI. The investigators noted a 30-day mortality rate of 6.1% and an implantation success rate of 96.7%. Regarding some of the complications defined by Valve Academic Research Consortium-2 (VARC-2 criteria), stroke was described in 1%, procedure-related mortality in 3.1%, vascular complications in 3.4%, and acute kidney injury in 9.5% of the patients (24). The register also included patients who had no contraindication for TF access. Further investigations from the register even supported that TAo access can not only be used as a last resort strategy (30). In 2018, the registry reported its 1-year follow-up data. The authors observed a mortality rate of 19.1%, a stroke rate of 4.0%, and an acute kidney injury rate of 14.7%. These results seem to be comparable to those of other non TF access routes (31). Nevertheless, the registry does not directly compare TAo-outcomes with those of other alternative access routes. On the basis of this data, determination of the preferred nontransfemoral technique for TAVI is extremely difficult. In the following paragraphs, a short summary of the existing literature comparing nontransfemoral access routes with TAo access is provided. An overview of these studies is summarized in Table 1.

**Table 1:** Overview of selected studies comparing transaortic with other alternative access routes.

**References**	**Design**	**Approach**	**Patient number**	**Age**	**STS PROM (% or OR)**	**Perioperative mortality** **(% or OR)**	**Stroke (% or OR)**	**Major bleeding complication (% or OR)**	**Acute kidney injury (% or OR)**	**Vascular complication** **(% or OR)**	**Pacemaker implantation (% or OR)**
***Transapical vs. transaortic***											
Thourani (32)	Multicenter registry	TA TAo	4,085 868	82.6 ± 6.8 83.6 ± 6.8	8.8%^*^ 7.4%^*^	6.8% 8.1%	2.1% 2.5%	N/A	38.8% 39.6%	0.3% 0.3%	N/A
Ribeiro (10)	Single-center retrospective observational study	TA TAo	206 45	78 ± 8 81 ± 7	7.4% 7.0%	9.2% 11.2%	4.0% 4.5	22.8 33.3	N/A	7.0% 9.1%	9.2% 11.2%
Dunne (33)	Meta analysis	TA TAo	9,619 342	79.9 80.7	N/A	9.7% 7.9%	2.1 0.9	9.4 8.0	N/A	N/A	5.9% 5.5%
Arai (34)	Single-center retrospective observational study	TA TAo	42 289	81.3 83.7	7.1% 5.8%	14% 9%	5 2	7 6	31%^*^ 13%^*^	N/A	7% 10%
***Transaxillary vs. transaortic***											
Fiorina (18)	Multicenter registry	TAx TAo	147 95	83 ± 5 82 ± 6	6% [4–12]^*^ 10% [5–4]^*^	5% 9%	1.4% 1.1%	4% 5%	22%^*^ 36%^*^	14%^*^ 5.3%^*^	34%^*^ 13%^*^
Lee (35)	Meta analysis	TAx TAo	2,136 2,236	80.0 82.7	8.9% 7.5%	5.7%^*^ 9.6%^*^	5.8%^*^ 2.6%^*^	8.5% 14.5%	8.2% 11.7%	3.8% 4.4%	20.1%^*^ 12.3%^*^
Takagi (15)	Meta analysis	TAx TAo	965 335	N/A	N/A	OR 0.48 [0.25–0.92]^*^	OR 2.54 [0.63–10.18]	OR 1.73 [0.88–3.39]	OR 0.49 [0.28–0.86]^*^	OR 1.43 [0.34–6.05]	OR 3.08 [1.96–4.84]^*^
***Transcarotid vs. transaortic***											
Thourani (16)	Single-center retrospective observational study	TC TAo	11 35	68.9 83.8	17.1% 11.5%	0% 11.4%	0% 2.9%	N/A	N/A	N/A	N/A
Allen (19)	Single-center retrospective observational study	TC TAo	84 33	78.9 78	9% 10%	2.4% 6.1%	2.4% 3%	N/A	N/A	3.6% N/A	8.3% N/A
Damluji (36)	Multicenter registry	TC TAo	43 67	81 84	6.9% 7.2%	8% 18%	2% 6%	5% 7%	2% 4%	5% 9%	N/A
Chamandi (37)	Multicenter registry	TC Tao	101 N/A	80.4 N/A	6.6% N/A	5% N/A	2.9% N/A	4% N/A	0% N/A	3% N/A	6.9% N/A

*OR, odds ratio; N/A, not available*.

* *statistically significant*.

## Transapical vs. Transaortic TAVI

In an article by the Society of Thoracic Surgeons (STS/American College of Cardiology Transcatheter Valve Therapy Registry), TA access (*n* = 4,085) was compared with TAo access (*n* = 868). Patients undergoing TAo TAVI had a higher STS PROM (8.8 vs. 7.4%, *p* < 0.001) and an increased risk of 30-day mortality (10.3 vs. 8.8%, *p* = 0.006) and 1-year mortality (30.3% vs. 25.6%). However, after risk adjustment, no significant difference between TA and TAo access was found regarding mortality, stroke, or readmission for as long as 1 year after TAVI (32).

Ribeiro et al. investigated the impact of access routes on myocardial injury (TAo *n* = 45, TA *n* = 206). They demonstrated a significantly increased myocardial injury in the TA approach. Furthermore, TA approach was associated with reduced left ventricular function improvement and lower-early and midterm-survival rates. The authors concluded that the higher degree of myocardial injury in the TA group led to the inferior clinical outcome (10).

In a meta-analysis of 60 articles comparing 9,619 TA patients with 342 TAo patients, the 30-day mortality rate was 7.9 vs. 9.7%. The stroke rate was calculated as 0.9 vs. 2.1%. In this large patient population, the authors observed no significant differences in all analyzed outcome parameters (33).

In 2016, Arai et al. compared the feasibility and safety of TF (*n* = 467), TAo (*n* = 289), and TA access (*n* = 42) routes. TF access was superior regarding kidney injury (TAo 13 vs. TF 5%, *p* < 0.001) and life-threatening bleeding (TAo 6 vs. TF 3%, *p* = 0.021). TAo access was better than TF access regarding the rate of permanent pacemaker implantation (TAo 10 vs. TF 16%, *p* = 0.032). Compared with TA access, TAo showed better results for acute kidney injury (TAo 13 vs. TA 31%, *p* = 0.003). There was no difference in the 30-day (TAo 9 vs. TA 14%, *p* = 0.283; TF 5 vs. TAo 9%, *p* = 0.057) and 1-year mortality (log rank: TF vs. TAo, *p* = 0.067; TAo vs. TA, *p* = 0.154) (34).

Nevertheless, the comparison between TA and TAo access is probably the most important as both procedures provide the possibility to avoid the aortic arch. Especially patients with a respiratory dysfunction or a poor left ventricular function can benefit from TAo access (14). Moreover, TAo approach utilizes an upper ministernotomy, which is well-tolerated by patients and familiar among surgeons and does not require a long learning curve (38).

All these investigations were performed retrospectively and consequently, and different access routes are linked to a selection bias, resulting in differences regarding the preoperative risk characteristics.

## Transaxillary vs. Transaortic TAVI

Compared to the TAo approach, the TAx approach offers advantages in terms of the integrity of the thorax. In addition, atherosclerosis tends to affect the supra-aortic branches less. Consequently, this access route remains available despite the peripheral arterial disease. Investigators from the Italien CoreValve Registry have compared TAx to TAo access in 242 patients. They summarize that despite the higher risk profile in the TAo group, there was no difference in 30-day mortality (TAo 9 vs. TAx 5%, *p* = 0.5). In addition, there was a significant reduction in the rate of permanent pacemaker implantation (TAo 13 vs. TAx 34%, *p* = 0.017) and a trend toward fewer paravalvular leaks (TAo 6 vs. TAx 14%, *p* = 0.07). On the other hand, TAo was associated with a longer hospital stay (TAo 10 days vs. TAx 8 days, *p* = 0.001) and a higher rate of acute kidney injury (TAo 36 vs. TAx 22%, *p* = 0.017) (18). In a meta-analysis from Lee et al., 31 studies with 4,372 patients were included. In this patient population, TAo had a lower STS score (TAo 7.5 vs. TAx 8.9%), lower risk of stroke (TAo 2.6 vs. TAx 5.6%, *p* < 0.001), and a lower rate of permanent pacemaker implantation (TAo 12.3 vs. 20.1%, *p* = 0.009). However, the 30-day mortality rates were significantly higher in the TAo group (TAo 9.6 vs. 5.7%, *p* < 0.001) (35). Another meta-analysis conducted by Takagi et al. compared TAx with TF, TA, and TAo access. Focusing on the comparison of TAx and TAo approach, the early mortality was lower in TAx TAVI [OR 0.48 (0.25–0.92), *p* = 0.03]. In the midterm all-cause mortality, no difference was observed [OR 0.94 (0.65–1.35), *p* = 0.73]. Once again, the permanent pacemaker implantation rate was higher in the TAx approach group [OR 3.08 (1.96–4.84), *p* < 0.001] (15).

Due to the lack of randomized controlled trials, all these investigations and meta-analyses are based on the results of secondary endpoints of a retrospective analysis. Therefore, this analysis may help to create a hypothesis rather than provide a conclusion. However, in these data TAx TAVI is a notable alternative for nontransfemoral access with a slightly lower early mortality rate compared to the TAo access route.

## Transcarotid vs. Transaortic TAVI

The first successful TC TAVI was performed in 2010 (39). It provides an additional option for a closed chest nontransfemoral TAVI.

In the French TAVI registry, 314 patients underwent TC TAVI procedure. The procedural success rate was high (97%) with a low 30-day mortality (3.2%) and even lower cerebrovascular ischemic event (1.6%) (40). Debry et al. investigated the impact of general (*n* = 122) or local anesthesia (*n* = 52) in TC TAVI procedure. They described a significantly higher rate of stroke in general anesthesia (5.7 vs. 0%, *p* < 0.001). In their opinion, the difference between both groups is most likely caused by the learning curve, the start of the TC access program under general anesthesia (41). Thourani et al. published a small patient cohort comparing TAo (*n* = 35) and TC (*n* = 11) access routes. There was no difference observed, but the study is limited by the small cohort (16). Allen et al. demonstrated in a retrospective study comparing TC (*n* = 84), TA (*n* = 48), and TAo (*n* = 33) the shorter length of stays (TC 3.0 days vs. TA 6.5 days vs. TAo 7.0 days; *p* < 0.001), fewer transfusions (TC 4.8 vs. TA 25 vs. TAo 24.2%; *p* < 0.001), and a better 2-year survival (TC 88.4 vs. 79.2 vs. TAo 63.6%; log rank *p* = 0.004) for the TC approach compared with the TA and TAo. The stroke rate did not differ between the groups. Despite a small number of patients included in this analysis and the retrospective study design, preoperative characteristics are highly comparable (19). Data from a multicenter registry including all alternative access routes (*n* = 172), TAo (*n* = 67), were associated with an 18% perioperative mortality rate followed by an 8% mortality rate in TC (*n* = 43) access (*p* = 0.08) (36). In a propensity score-matched analysis comparing TC (*n* = 94) with transthoracic TAVR (TAo and TA; *n* = 163), a lower rate of major bleeding (TC 4.3% vs. transthoracic TAVR 19.9%, *p* = 0.002), atrial fibrillation (TC 3.2 vs. transthoracic TAVR 19%, *p* = 0.002), and acute kidney injury (TC 0 vs. transthoracic TAVR 12.1%, *p* = 0.002) were observed in the TC access group. Nevertheless, 30-day mortality rates (TC 2.1 vs. transthoracic TAVR 4.6%, *p* = 0.37) were similar (37).

All these encouraging results are obtained from retrospective analysis with small patient numbers. Nevertheless, these data showed that TC access is safe and feasible without excessive risk of embolic stroke or vascular complications.

## Conclusion and Future Perspectives

Without a doubt, the TF approach is the gold standard in TAVI. Nevertheless, the TAo approach has proven to be a safe and efficient alternative when TF access is not suitable. In the currently available literature, supra-aortic access routes such as TAx and TC have shown very promising results when compared with TAo access. The benefit of preserving the thoracic integrity resulted in a shorter length of stay and fewer bleeding complications. On the other hand, paravalvular leakage and permanent pacemaker implantation occurred less frequently in the TAo group. However, 30-day mortality rates were lower in the supra-aortic approach TAVI.

The studies comparing TAo and TA TAVI did not indicate a clear superiority of one approach. Interestingly, TAo was shown to be associated with a lower risk for myocardial injury and better outcomes in terms of acute kidney injury. Small patient cohorts and differences in preoperative comorbidities are the major limitations of these retrospective studies, which make data interpretation very challenging.

Despite the fact that the individual anatomy receives little attention in these studies, it is the key factor for the choice of the optimal alternative access route. In the presence of calcification in the supra-aortic branches and the aortic arch, TAx and TC approaches are not feasible. In this patient cohort, TAo TAVI remains a valuable option with some advantages over the TA access route, especially in patients with poor left ventricular function and chronic lung disease.

Due to ongoing technical improvements in the TF access route, the need for alternative routes has decreased over the last 10 years. To provide a tailor-made therapy for all patients, a future concept should be implanted stratifying standard patients for TF TAVR for treatment in a low-volume center and transferring more advanced cases to specialized TAVI teams with expertise in all alternative access routes.

## Author Contributions

LS, CK, and NB contributed to conception and design of the review. LS wrote the first draft of the manuscript. NB and JD contributed to manuscript revision. GFe and AM contributed to figure design. All authors contributed to manuscript revision, read, and approved the submitted version.

## Conflict of Interest

The authors declare that the research was conducted in the absence of any commercial or financial relationships that could be construed as a potential conflict of interest.

## Publisher's Note

All claims expressed in this article are solely those of the authors and do not necessarily represent those of their affiliated organizations, or those of the publisher, the editors and the reviewers. Any product that may be evaluated in this article, or claim that may be made by its manufacturer, is not guaranteed or endorsed by the publisher.
